# Massive Multinodular Head and Neck Recurrence of Parotid Gland Pleomorphic Adenoma: A Case Report

**DOI:** 10.1155/2014/914021

**Published:** 2014-06-22

**Authors:** Pierre Philouze, Nicolas Sigaux, Anne Frédérique Manichon, Jean-Christian Pignat, Marc Poupart

**Affiliations:** ^1^Service d'ORL, Hôpital de la Croix-Rousse, 103 Grande rue de la Croix-Rousse, 69317 Lyon Cedex 4, France; ^2^Service de Radiologie, Hôpital de la Croix-Rousse, 103 Grande rue de la Croix-Rousse, 69317 Lyon Cedex 4, France

## Abstract

*Introduction*. The optimal initial management of parotid pleomorphic adenomas reduces the risk of recurrence and malignant transformation. Surgery of recurrence can be difficult in multinodular disseminated forms. *Case Report*. A 67-years-old patient was referred for management of a large multifocal recurrence of a pleomorphic adenoma operated on 23 years ago. The clinical and radiological assessment found parapharyngeal, infratemporal, and prestyloid invasion, with nodules in the sternocleidomastoid muscle. Excision by transmandibular approach was performed. The pathologist found a multinodular recurrent pleomorphic adenoma without criteria of malignancy. Postoperative radiotherapy was performed. *Discussion*. Multinodular forms and incomplete resections are the most important factors that are thought to predispose to recurrence. A precise analysis of the extension by preoperative MRI is essential. Adjuvant radiotherapy can be given in these recurrent multifocal forms.

## 1. Introduction 

The benign pleomorphic adenoma is the most common parotid tumor. Optimal initial management is essential to reduce the risk of recurrence and malignant transformation. We present the case of a large multinodular recurrence of a pleomorphic adenoma. We describe and discuss the diagnostic, management, surgical, and postoperative care.

## 2. Case Presentation

A patient of 67 years was referred for management of recurrent parotid pleomorphic adenoma. Surgical management of a pleomorphic adenoma of the deep lobe of the right parotid gland was made 23 years ago. Pathological findings confirmed the benign nature of the tumor. No additional treatment had been initiated.

Clinically, a multifocal recurrence was found in the parapharyngeal space, the lower part of the right parotid space, and along the cervical scar. It showed no facial paralysis or pain or inflammation in the lesion area.

Fine-needle aspiration showed arguments for pleomorphic adenoma but no signs of malignancy.

**Figure 1:**
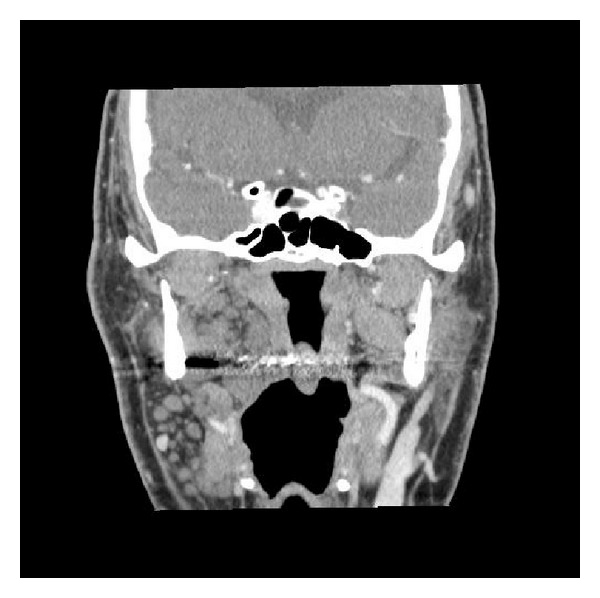
Preoperative tomodensitometry.

Cervicofacial tomodensitometry found multiple nodules in parapharyngeal and prestyloid spaces and in the sternocleidomastoid muscle (Figure 1).

**Figure 2:**
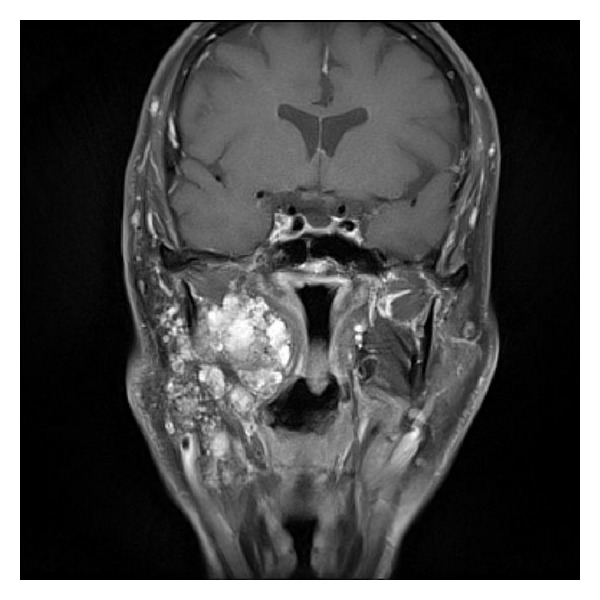
Preoperative MRI T1 Fat Sat gadolinium.

**Figure 3:**
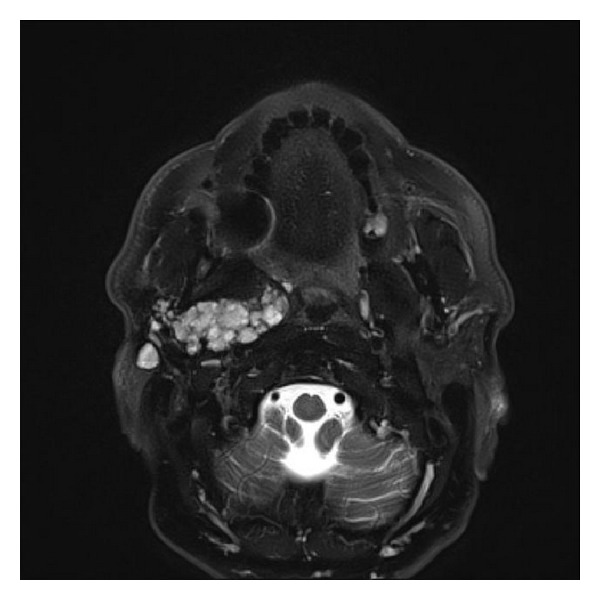
Preoperative MRI T2 Fat Sat.

Additional MRI found a multilobulated lesion of the right parotid gland, hypointense on T1 and hyperintense on T2. This lesion infiltrated the superficial lobe and is extended to the deep lobe of 61 × 30 × 85 mm. It infiltrated inside the infratemporal space to the parapharyngeal space. It repressed the medial pterygoid muscle. Backward it repressed the digastric muscle. The external carotid artery was walking in contact with the lesion. The deepest portions came in contact with the internal carotid artery. The facial nerve passed into the tumor. And there were multiple nodular cystic lesions in fatty areas below the parotid gland in front of and behind the sternocleidomastoid muscle. The uppermost lesion was located just below the external auditory canal (Figures 2 and 3).

On the MRI, curves of infusion and appearance of these lesions in diffusion favored pleomorphic adenoma. Revision surgery was indicated. Given the size of the tumor, a transmandibular approach has been decided on during surgery. It consisted of a monobloc resection of the tumor. In order to excise all the cervical nodules disseminated into the sternocleidomastoid muscle and between intern jugular vein and carotid artery, a lymph node cervical dissection was necessary. The specimen measured 9 × 6 × 3 cm.

Multinodular recurrence was invading prestyloid and parapharyngeal spaces, with nodules invading the anterior pillar of the tonsil and also coming into contact with the maxillary tuberosity. Multiple nodules were found in the sternocleidomastoid muscle so that a part had to be resected. The facial nerve was identified, dissected, and preserved. An electromyographic monitoring system was used. Given the loss of substance, a pectoralis major flap was necessary for closure.

The pathological results showed multinodular recurrence of a pleomorphic adenoma:

- in the parapharyngeal space measuring a major axis of 8 cm,
- above spinal accessory nerve, along the jugular vein and the carotid artery, below the digastric muscle, in the subcutaneous space, and in the tonsillar fossa. The upper part was in contact with the maxillary tuberosity and nodules were found around the stylomastoid foramen. The superficial parotid was invaded.

No microscopic evidence of malignant transformation was found and absence of lymph node metastasis of 18 nodes was analyzed.

Given the recurrent nature and large multinodular tumor, adjuvant radiotherapy was decided on. One year after surgery and radiotherapy, no recurrence occurred.

## 3. Discussion

Surgical resection is the standard treatment of parotid pleomorphic adenoma. Several studies agree on the risk factors for recurrence: an incomplete initial surgical management with capsular rupture or enucleation, multinodular histological types, young age, and a history of recurrent adenoma [1, 2].

The surgical management of recurrent pleomorphic adenoma is often difficult. Indeed, multifocal recurrences can lead to large resections often carrying the facial nerve. The evaluation of the size of the recurrence and its location is an important step in the management. This is to inform the patient about the surgical procedures and risks and to adapt the surgical approach.

MRI remains the gold standard in the diagnosis of primary or recurrent tumors of salivary glands [3]. Dynamic analysis of the tumor contrast and diffusion imaging with measurement of apparent diffusion coefficient are valuable tools for diagnostic [4]. Recurrent pleomorphic adenoma is often multifocal, involving both the tumor bed and the adjacent spaces. These lesions, often small nodules with diameter less than one centimeter, appear strongly hyperintense in T2 sequences, which are easy to identify, according to some authors [5].

In the case of our patient, MRI has been used to characterize the multinodular lesion with parapharyngeal and infratemporal extension. But it helped also in locating nodules in fatty areas below the parotid and forward and backward of sternocleidomastoid muscle. The uppermost lesion is located just below the external auditory meatus.

These findings help the surgeon in the choice of the surgical approach in order to make an optimal cervical exploration and to excise intramuscular nodules. For the main lesion, a transmandibular approach was necessary [6]. Indeed, it is usually appropriate to choose submandibular approach for parapharyngeal tumors, to avoid the mandibulotomy. However, in this case, multifocal involvement and contact with the maxillary tuberosity imposed a transmandibular approach. The exposition was perfect and vascular risks were mastered.

The role of radiotherapy after recurrence of pleomorphic adenoma remains controversial. For some authors, it is indicated in recurrences, especially in multinodular forms [7, 8]. Moreover, the prior surgical control seems necessary before adjuvant radiotherapy [9]. This is covered in the recent review of the French Society for Radiotherapy and Oncology [10].

## 4. Conclusion

Resection of recurrent multifocal pleomorphic adenoma must be as complete as possible and MRI should be performed preoperatively to localize nodules. Adjuvant radiotherapy remains debated but may be useful in such forms of recurrences.
